# Probable Hypophysitis Recognized More Than Two Years After Pembrolizumab Discontinuation

**DOI:** 10.7759/cureus.117267

**Published:** 2026-09-30

**Authors:** Bibek Adhikari, Sushil Rayamajhi, Sneha Dhakal

**Affiliations:** 1 Hospital Medicine, Faith Regional Health Services, Norfolk, USA

**Keywords:** central adrenal insufficiency, central hypothyroidism, delayed immune-related adverse event, hypophysitis, hypopituitarism, immune checkpoint inhibitor, pembrolizumab, pituitary enlargement

## Abstract

Immune checkpoint inhibitor-associated hypophysitis is an uncommon but clinically important endocrine immune-related adverse event. We report a 70-year-old man with a history of non-small cell lung cancer previously treated with approximately two years of pembrolizumab, with his last dose administered approximately 28 months before presentation. He developed progressive fatigue, anorexia, hypotension, functional decline, and confusion and was subsequently hospitalized with multifocal pneumonia and septic shock. Although the infection and hemodynamic instability improved, encephalopathy, weakness, poor oral intake, and dysphagia persisted. Brain magnetic resonance imaging demonstrated new pituitary enlargement compared with imaging obtained approximately three months earlier. Dedicated pituitary imaging showed enlargement measuring approximately 1.5 × 2.0 cm without definite differential enhancement to suggest a pituitary adenoma. Endocrine testing demonstrated a cortisol level of 3.3 µg/dL with an inappropriately low adrenocorticotropic hormone level of 6.5 pg/mL, free thyroxine of 0.36 ng/dL with thyroid-stimulating hormone of 2.0 µIU/mL, suppressed gonadotropins, and testosterone <10 ng/dL, supporting central adrenal insufficiency and central hypothyroidism with marked gonadal-axis suppression. Cerebrospinal fluid cytology and paraneoplastic studies were unrevealing. The patient was treated with hydrocortisone before thyroid hormone replacement and subsequently showed progressive improvement in mental status, oral intake, and participation in rehabilitation. This case highlights that pituitary immune-related adverse events may be recognized long after pembrolizumab discontinuation and emphasizes the importance of considering prior immune checkpoint inhibitor exposure in patients with unexplained endocrine and constitutional decline.

## Introduction

Immune checkpoint inhibitors have substantially improved outcomes in several malignancies but can produce immune-related adverse events involving nearly every organ system. Endocrine toxicities are particularly challenging because symptoms such as fatigue, anorexia, weakness, hypotension, hyponatremia, and cognitive changes are nonspecific and may be attributed to malignancy, infection, treatment effects, or deconditioning [1,2].

Hypophysitis is inflammation of the pituitary gland that can result in deficiency of one or more pituitary hormones. Immune checkpoint inhibitor-associated hypophysitis is thought to result from immune-mediated inflammation and injury of pituitary cells. However, its precise pathophysiology remains incompletely understood. Inflammatory infiltration and edema may account for pituitary enlargement during the inflammatory phase. Hypophysitis is more commonly associated with cytotoxic T-lymphocyte-associated protein 4 inhibition than with programmed cell death protein 1 (PD-1) or programmed death-ligand 1 (PD-L1) inhibition. The clinical phenotype also differs by drug class. PD-1/PD-L1-associated hypophysitis frequently presents with adrenocorticotropic hormone (ACTH) deficiency and may occur with a normal pituitary magnetic resonance imaging (MRI) study [1,3]. In contrast, marked pituitary enlargement with dysfunction of several anterior pituitary axes is less typical.

Most reported cases develop during treatment or within months of immune checkpoint inhibitor exposure. Delayed immune-related adverse events occurring after treatment discontinuation are increasingly recognized [4,5]. Pembrolizumab is a PD-1 inhibitor. We describe probable hypophysitis with central adrenal insufficiency, central hypothyroidism, and marked gonadal-axis suppression recognized approximately 28 months after the patient's final pembrolizumab exposure.

## Case presentation

A 70-year-old man with a history of stage III non-small cell lung cancer (NSCLC) previously treated with pembrolizumab, metastatic prostate cancer with osseous metastases, and recently resected stage I colon cancer was admitted with fever, hypotension, and worsening confusion.

His NSCLC had been treated with approximately two years of pembrolizumab together with stereotactic radiation for oligoprogressive pulmonary disease. Pembrolizumab had been discontinued approximately 28 months before the current presentation, and his lung cancer had subsequently been managed with surveillance. His prostate cancer had previously been treated with androgen-directed therapy; enzalutamide was listed as an outpatient medication but was held during this hospitalization.

According to his family, his functional status had progressively declined during the months preceding hospitalization. He developed worsening fatigue, poor appetite, intermittent hypotension, episodes of hypothermia, impaired temperature regulation, and increasing confusion. He became increasingly dependent on activities of daily living and eventually required wheelchair assistance. At presentation, he was febrile and hypotensive despite intravenous fluids and required vasopressor support. He was admitted to the intensive care unit with septic shock attributed to multifocal pneumonia. Computed tomography demonstrated multifocal pulmonary infiltrates, greatest in the right lower lobe, together with bilateral pleural effusions. He received broad-spectrum antimicrobial therapy and completed a seven-day antibiotic course.

His vasopressor requirement resolved rapidly, and his acute kidney injury improved. Despite treatment of the infection and stabilization of his hemodynamics, he continued to have significant encephalopathy, profound weakness, poor oral intake, and dysphagia. Due to persistent mental status changes and his oncologic history, neurologic and oncologic evaluation was pursued. The differential diagnosis included persistent toxic-metabolic encephalopathy, metastatic or leptomeningeal disease, paraneoplastic or autoimmune encephalopathy, and seizure. Electroencephalography demonstrated continuous delta slowing consistent with moderate nonspecific encephalopathy, although the recording was limited by motion and muscle artifact.

Lumbar puncture was performed. Cerebrospinal fluid cytology was negative for malignant cells. Serum and cerebrospinal fluid paraneoplastic studies and West Nile virus testing were negative. Matching oligoclonal bands were present in serum and cerebrospinal fluid, favoring a systemic inflammatory response rather than an isolated intrathecal process.

Brain magnetic resonance imaging (MRI) demonstrated interval enlargement of the pituitary gland compared with imaging obtained approximately three months earlier. A dedicated pituitary-protocol MRI subsequently demonstrated pituitary enlargement measuring approximately 1.5 × 2.0 cm in the anteroposterior and transverse dimensions. Dynamic imaging showed no definite differential enhancement to suggest a pituitary adenoma. There was minimal superior displacement of the optic chiasm, and the infundibulum was not distinctly visualized (Figure 1). Assessment of headache, visual symptoms, polyuria, and polydipsia was limited by the patient's encephalopathy; these symptoms were not reported during the available clinical assessments. Formal visual field testing was not performed.

**Figure 1:**
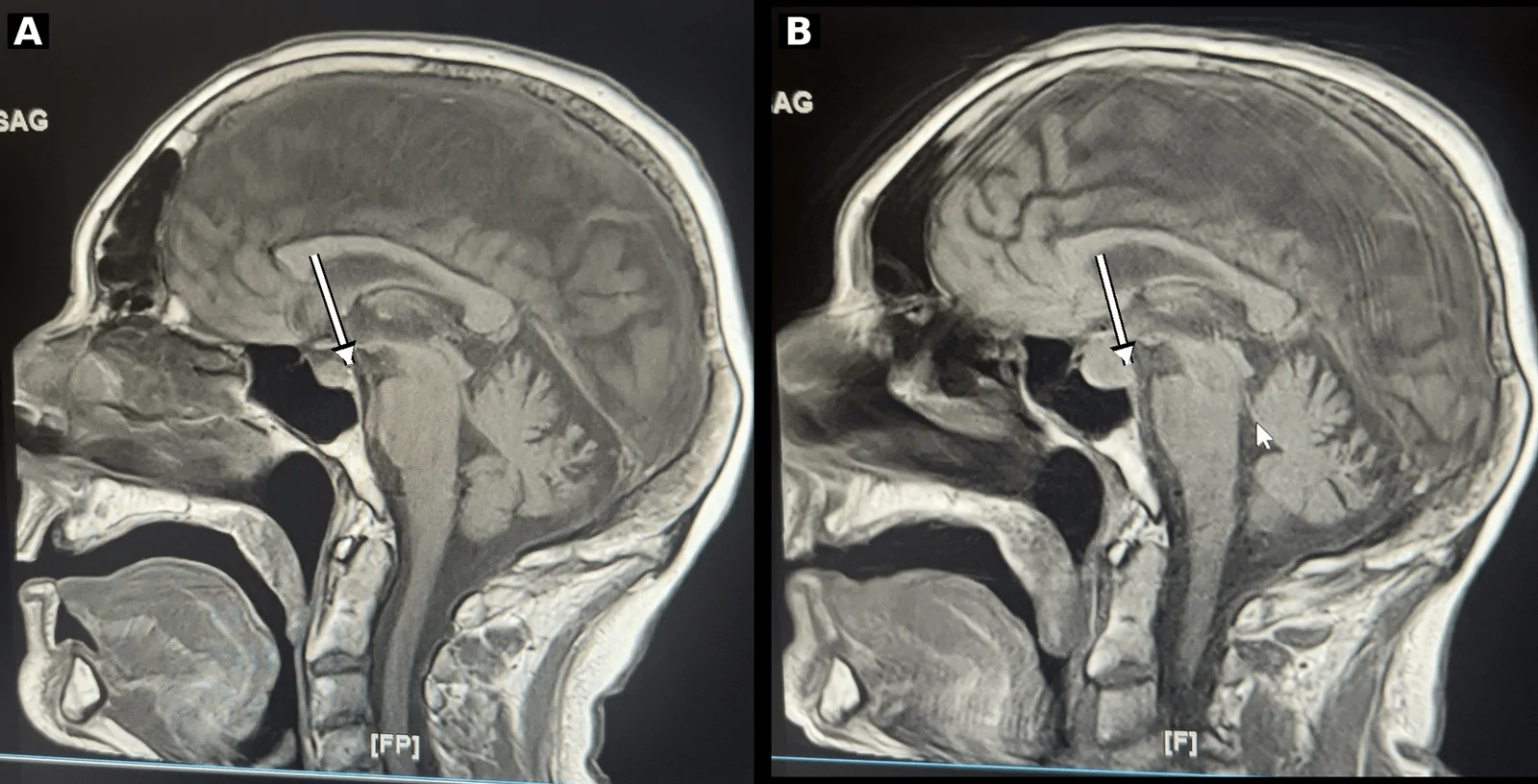
Interval Development of Pituitary Enlargement Sagittal magnetic resonance images obtained on July 22, 2025 (A), and October 23, 2025 (B), demonstrate interval enlargement of the pituitary gland. Arrows indicate the pituitary gland. The displayed sagittal images demonstrate the interval change in pituitary size. On the complete dedicated pituitary magnetic resonance imaging examination performed on October 23, 2025, the gland measured approximately 1.5 × 2.0 cm, with no definite differential enhancement on dynamic imaging to suggest a pituitary adenoma and with minimal superior displacement of the optic chiasm.

A comprehensive metabolic panel showed mild hyponatremia with a sodium concentration of 133 mEq/L and a glucose concentration of 85 mg/dL. Pituitary hormone testing was obtained before glucocorticoid therapy was initiated. No pre-illness pituitary hormone measurements were available for comparison. Endocrine evaluation demonstrated abnormalities involving several anterior pituitary axes (Table 1).

**Table 1:** Endocrine laboratory evaluation Pituitary hormone testing was obtained before initiation of glucocorticoid therapy. The baseline serum cortisol was collected at 05:05. A standard 250-µg cosyntropin stimulation test subsequently produced cortisol concentrations of 16.6 µg/dL at approximately 30 minutes and 21.6 µg/dL at approximately 60 minutes. Reference ranges are those reported by the performing laboratories. For FSH, the performing laboratory reported only an upper reference limit for men older than 70 years. The DHEA-S interval shown is the reference interval reported by the performing laboratory. The IGF-1 concentration fell within the laboratory-reported concentration reference interval, whereas its age- and sex-adjusted Z-score was −3.1; the Z-score was therefore interpreted separately and cautiously in the context of advanced age, malnutrition, and acute systemic illness. ACTH: adrenocorticotropic hormone; DHEA-S: dehydroepiandrosterone sulfate; FSH: follicle-stimulating hormone; IGF-1: insulin-like growth factor 1; LH: luteinizing hormone; T4: thyroxine; TSH: thyroid-stimulating hormone.

Test	Result	Reference Range
Cortisol, serum (baseline, 05:05)	3.3 µg/dL	Morning: 4.0–22.0 µg/dL
ACTH	6.5 pg/mL	7.2–63.3 pg/mL
Cortisol, ~30 minutes after cosyntropin	16.6 µg/dL	—
Cortisol, ~60 minutes after cosyntropin	21.6 µg/dL	—
Free T4	0.36 ng/dL	0.8–1.8 ng/dL
TSH	2.0 µIU/mL	0.550–4.780 µIU/mL
LH	<0.1 mIU/mL	Male >69 years: 2.0–26.0 mIU/mL
FSH	<0.3 mIU/mL	Male >70 years: <87.0 mIU/mL
Total testosterone	<10 ng/dL	250–950 ng/dL
IGF-1	26 ng/mL (Z-score −3.1)	22–221 ng/mL
Prolactin	15.7 ng/mL	2.6–13.1 ng/mL
DHEA-S	<5 µg/dL	80–560 µg/dL

The combination of a serum cortisol level of 3.3 µg/dL with an adrenocorticotropic hormone (ACTH) level of 6.5 pg/mL supported central adrenal insufficiency because the ACTH concentration was unusually low for the degree of hypocortisolemia. A standard 250-µg cosyntropin stimulation test increased serum cortisol from a baseline of 3.3 µg/dL to 16.6 µg/dL at approximately 30 minutes and 21.6 µg/dL at approximately 60 minutes. Although this demonstrated preserved adrenal responsiveness to exogenous ACTH, a normal standard-dose cosyntropin response does not exclude relatively recent central adrenal insufficiency because adrenal responsiveness may remain intact before prolonged endogenous ACTH deficiency causes adrenal cortical atrophy [6].

Free thyroxine (T4) was markedly reduced at 0.36 ng/dL with a thyroid-stimulating hormone (TSH) concentration of 2.0 µIU/mL. The TSH concentration was physiologically normal in the setting of profound hypothyroxinemia and was therefore consistent with central hypothyroidism. Luteinizing hormone (LH) was <0.1 mIU/mL, follicle-stimulating hormone (FSH) was <0.3 mIU/mL, and total testosterone was <10 ng/dL, demonstrating marked gonadal-axis suppression. Interpretation of these findings was complicated by his prostate cancer treatment history and acute systemic illness. The insulin-like growth factor 1 (IGF-1) levels were at 26 ng/mL, but the age- and sex-adjusted Z-score was −3.1. This finding was interpreted cautiously because advanced age, malnutrition, and acute systemic illness may affect the growth hormone/IGF-1 axis. Prolactin was mildly elevated at 15.7 ng/mL. Dehydroepiandrosterone sulfate (DHEA-S) was markedly reduced at <5 µg/dL.

In the setting of newly developed pituitary enlargement, central adrenal insufficiency, central hypothyroidism, and other pituitary hormone abnormalities, Endocrinology favored hypophysitis. Pituitary metastasis remained an important alternative diagnosis because of the patient's oncologic history, although the radiographic appearance was not considered typical of metastatic disease. No tissue diagnosis was obtained.

Treatment was initiated with intravenous hydrocortisone 50 mg every six hours. As central adrenal insufficiency and central hypothyroidism were present concurrently, glucocorticoid replacement was started before thyroid hormone replacement. Intravenous levothyroxine was subsequently initiated and later transitioned to oral levothyroxine 100 µg daily. Hydrocortisone was gradually tapered to oral replacement dosing of 20 mg in the morning and 10 mg later in the day, together with instructions for stress dosing during intercurrent illness.

Over the following days, his mental status gradually improved. He became more awake and interactive, followed commands more consistently, and participated increasingly in rehabilitation. Oral intake also improved. As his infection, nutritional status, and other medical problems were improving concurrently, the clinical response could not be attributed solely to hormone replacement. Nevertheless, the treating teams considered central adrenal insufficiency and hypothyroidism important contributors to his prolonged encephalopathy and functional decline. 

His hospitalization was also complicated by severe malnutrition and dysphagia. A modified barium swallow demonstrated severe oral and moderate pharyngeal dysphagia with delayed swallow initiation and significant aspiration risk. As cognition improved, he was eventually able to tolerate a modified oral diet and oral medications. He also developed a brief episode of torsades de pointes in the setting of marked bradycardia, potassium of approximately 3.0 mEq/L, and magnesium of 1.8 mg/dL. Echocardiography demonstrated preserved systolic function. Electrolyte abnormalities were corrected, and the arrhythmia did not recur. The event was considered multifactorial, with hypokalemia and hypomagnesemia representing major contributors and central hypothyroidism and bradycardia potentially increasing susceptibility.

By discharge, the pneumonia and septic shock had resolved, his encephalopathy had substantially improved, and he was tolerating oral medications and nutrition. He was discharged to a skilled nursing facility for rehabilitation on chronic hydrocortisone and levothyroxine with outpatient endocrinology follow-up.

## Discussion

Immune checkpoint inhibitor (ICI)-associated hypophysitis is an uncommon but clinically important endocrine immune-related adverse event. Its phenotype varies according to the checkpoint pathway targeted. Hypophysitis associated with cytotoxic T-lymphocyte-associated protein 4 (CTLA-4) blockade more commonly produces pituitary enlargement and multiple pituitary hormone deficiencies, whereas programmed cell death protein 1 (PD-1) inhibitor-associated hypophysitis frequently presents with isolated or predominant adrenocorticotropic hormone (ACTH) deficiency and may have normal pituitary imaging [1,3]. In the European Society of Endocrinology clinical practice guideline, ACTH deficiency was reported in approximately 97% of anti-PD-1-associated hypophysitis cases, whereas magnetic resonance imaging (MRI) abnormalities were considerably less frequent [1]. More recent analysis of 22 selected published pembrolizumab-associated cases similarly found ACTH deficiency to be the predominant hormonal abnormality; among cases with available MRI data, normal pituitary MRI was reported in 50% and pituitary enlargement in approximately 28% [7]. The present case is notable for radiographically documented interval pituitary enlargement accompanied by deficiencies involving several anterior pituitary axes rather than isolated ACTH deficiency.

The particularly important feature of this case is the prolonged interval between pembrolizumab discontinuation and recognition of pituitary dysfunction. Immune-related adverse events can develop after immunotherapy has been discontinued. Couey et al. introduced the term delayed immune-related events (DIRE) for new immune-related adverse events occurring at least 90 days after discontinuation of immunotherapy [4]. Delayed pituitary dysfunction has subsequently been described after programmed cell death protein 1/programmed death-ligand 1 (PD-1/PD-L1) blockade. Chen et al. identified cases of PD-1/PD-L1 inhibitor-associated hypophysitis developing four to 15 months after treatment discontinuation [5]. Individual reports have described ACTH deficiency four months after pembrolizumab discontinuation, adrenal insufficiency eight months after pembrolizumab cessation, and hypophysitis 22 months after discontinuation of durvalumab [8-10]. In the present case, the last pembrolizumab treatment had occurred approximately 28 months before presentation, representing an unusually prolonged interval to recognition. Although this temporal association cannot establish causality, it emphasizes that remote ICI exposure should remain clinically relevant when otherwise unexplained pituitary dysfunction develops.

The biochemical findings supported dysfunction at the pituitary level. The combination of a low serum cortisol concentration with an inappropriately low ACTH level was consistent with central adrenal insufficiency. Similarly, markedly reduced free thyroxine with a thyroid-stimulating hormone concentration that was not appropriately elevated supported central hypothyroidism. Suppressed gonadotropins with markedly reduced testosterone demonstrated marked gonadal-axis suppression; however, interpretation was complicated by his prostate cancer treatment history and acute systemic illness. The insulin-like growth factor 1 concentration was 26 ng/mL, within the laboratory's reported concentration reference range of 22-221 ng/mL. However, the age- and sex-adjusted Z-score was −3.1. This finding was interpreted cautiously because advanced age, malnutrition, and acute systemic illness may affect the growth hormone/insulin-like growth factor 1 axis. Taken together with the newly developed pituitary enlargement, however, the multiple anterior pituitary hormone abnormalities supported a clinically significant pituitary process.

An important diagnostic consideration in this patient was the apparently preserved response to standard-dose cosyntropin despite biochemical evidence of central adrenal insufficiency. The basal cortisol and ACTH measurements were obtained before glucocorticoid therapy, and the approximately sixty-minute stimulated cortisol concentration reached 21.6 µg/dL. A normal response to a 250-µg cosyntropin stimulation test does not reliably exclude recent or partial central adrenal insufficiency because the adrenal cortex may retain responsiveness to exogenous ACTH before prolonged endogenous ACTH deficiency results in adrenal atrophy [6]. Consequently, the basal cortisol and ACTH concentrations, clinical presentation, additional pituitary hormone abnormalities, and pituitary imaging were considered together rather than interpreting the stimulation test in isolation. As the preceding symptoms evolved over several months, their onset cannot be assumed to correspond to the onset of established ACTH deficiency.

Pituitary metastasis was an important alternative diagnosis because of the patient's oncologic history. However, the interval development of pituitary enlargement, associated pituitary hormone abnormalities, and history of pembrolizumab exposure favored hypophysitis clinically, while pituitary metastasis remained an unresolved alternative diagnosis. Cerebrospinal fluid cytology was negative for malignant cells, although negative cytology does not exclude an isolated pituitary metastasis. No pituitary biopsy was obtained; therefore, the diagnosis remained clinical rather than histopathologically confirmed. This distinction is important because neither pituitary enlargement nor endocrine dysfunction alone is specific for ICI-associated hypophysitis.

The patient's initial septic shock, severe malnutrition, and systemic illness also complicated interpretation of his presentation. Fatigue, hypotension, anorexia, weakness, and altered mental status are nonspecific findings that may occur with infection, malignancy, malnutrition, or adrenal insufficiency. In this case, persistence of profound weakness, encephalopathy, poor oral intake, and functional impairment despite improvement in infection and hemodynamics prompted further investigation. The subsequent identification of new pituitary enlargement and concordant hormonal abnormalities provided evidence of an additional endocrine process. His subsequent clinical improvement occurred in parallel with hormone replacement, treatment of infection, and nutritional and supportive care; therefore, the recovery cannot be attributed exclusively to endocrine therapy.

Management of central adrenal insufficiency takes priority when adrenal insufficiency and central hypothyroidism coexist because initiation of thyroid hormone before adequate glucocorticoid replacement may precipitate adrenal crisis [1,2,6]. Accordingly, this patient received intravenous hydrocortisone before levothyroxine was initiated and was subsequently transitioned to maintenance hormone replacement. He was discharged on hydrocortisone 20 mg in the morning and 10 mg later in the day, and oral levothyroxine 100 µg daily, with planned outpatient endocrinology follow-up. Post-discharge endocrine testing and follow-up pituitary imaging were not available for this report. Long-term endocrine follow-up is important because recovery of ICI-associated pituitary dysfunction, particularly ACTH deficiency, is uncommon [1]. An evidence-based duration for endocrine surveillance after ICI discontinuation has not been established; therefore, prior ICI exposure should remain clinically relevant when compatible symptoms develop after treatment cessation. In a recent analysis of 22 selected published pembrolizumab-associated hypophysitis cases, persistent endocrine dysfunction was reported in 95.5% despite clinical improvement following treatment [7].

ICI-associated hypophysitis is thought to result from immune-mediated inflammation of the pituitary following disruption of immune checkpoint tolerance. Inflammatory infiltration and edema may cause pituitary enlargement, while injury to hormone-producing cells can lead to persistent pituitary hormone deficiencies.

This case has several limitations. The diagnosis of hypophysitis was not histologically confirmed, and pituitary metastasis could not be excluded with absolute certainty. Acute infection, malnutrition, and severe systemic illness may also have influenced some endocrine measurements and clinical manifestations. Furthermore, the long interval after pembrolizumab discontinuation makes definitive attribution to prior immunotherapy difficult. Nevertheless, the combination of newly developed pituitary enlargement, central adrenal insufficiency, central hypothyroidism, other pituitary hormone abnormalities, and prior PD-1 inhibitor exposure supports ICI-associated hypophysitis as the favored clinical diagnosis. However, pituitary metastasis cannot be definitively excluded. The case illustrates the importance of maintaining awareness of possible endocrine immune-related adverse events even long after checkpoint inhibitor therapy has ended.

## Conclusions

Immune-related endocrine toxicity may be recognized long after immune checkpoint inhibitor therapy has ended. In this patient, probable hypophysitis with central adrenal insufficiency and central hypothyroidism was recognized approximately 28 months after the last pembrolizumab treatment and was accompanied by newly developed pituitary enlargement.

Prior immune checkpoint inhibitor exposure should remain in the differential diagnosis when patients with cancer develop otherwise unexplained fatigue, hypotension, hyponatremia, cognitive decline, or functional deterioration, even when immunotherapy was discontinued many months earlier. When central adrenal insufficiency and central hypothyroidism coexist, glucocorticoid replacement should precede thyroid hormone replacement.
